# A G1-lineage H9N2 virus with oviduct tropism causes chronic pathological changes in the infundibulum and a long-lasting drop in egg production

**DOI:** 10.1186/s13567-018-0575-1

**Published:** 2018-08-29

**Authors:** Francesco Bonfante, Eva Mazzetto, Claudia Zanardello, Andrea Fortin, Federica Gobbo, Silvia Maniero, Michela Bigolaro, Irit Davidson, Ruth Haddas, Giovanni Cattoli, Calogero Terregino

**Affiliations:** 1 Division of Comparative Biomedical Sciences, Istituto Zooprofilattico Sperimentale delle Venezie, Viale dell’Università, 10, 35020 Legnaro, Italy; 2 Histopathology Department, Istituto Zooprofilattico Sperimentale delle Venezie, Viale dell’Università, 10, 35020 Legnaro, Italy; 3 Avian Medicine Laboratory and Mycoplasmas Unit, Istituto Zooprofilattico Sperimentale delle Venezie, Viale dell’Università, 10, 35020 Legnaro, Italy; 4 Division of Avian Diseases, Kimron Veterinary Institute, 12, 50250 Bet Dagan, Israel; 5 Animal Production and Health Laboratory, Joint FAO/IAEA Division for Nuclear Applications in Food and Agriculture, International Atomic Energy Agency, Vienna International Centre, 100, 1400 Vienna, Austria

## Abstract

Since 1997, G1-lineage H9N2 avian influenza viruses have been circulating in Asia and later on in the Middle East, and they have been associated to mild respiratory disease, drops in egg production and moderate mortality in chickens, in particular in the presence of concurrent infections. In this study, we investigated the importance of the G1-lineage H9N2 A/chicken/Israel/1163/2011 virus as a primary pathogen in layers, analyzing its tropism and binding affinity for the oviduct tissues, and investigating the long-term impact on egg production. Besides causing a mild respiratory infection, the virus replicated in the oviduct of 60% of the hens causing different degrees of salpingitis throughout the organ, in particular at the level of the infundibulum, where the detection of the virus was associated with severe heterophilic infiltrate, and necrosis of the epithelium. Binding affinity assays confirmed that the infundibulum was the most receptive region of the oviduct. The drop in egg production was at its peek at 2 weeks post-infection (pi) (60% decrease) and continued up to 80 days pi (35% decrease). On day 80 pi, non-laying birds showed egg yolk peritonitis, and histopathological analyses described profound alteration of the infundibulum architecture, duct ectasia and thinning of the epithelium, while the rest of the oviduct and ovary appeared normal. Our results show that this H9N2 virus is a primary pathogen in layer hens, and that its replication in the infundibulum is responsible for acute and chronic lesions that limits the effective functionality of the oviduct, compromising the commercial life of birds.

**Electronic supplementary material:**

The online version of this article (10.1186/s13567-018-0575-1) contains supplementary material, which is available to authorized users.

## Introduction

Avian influenza viruses (AIV) of the H5, H7 and H9 subtypes represent an enormous commercial burden for the poultry sector, at a global level. Commercial, as well as political and sanitary issues related to the circulation of AIV, prompted policymakers and intergovernmental health organizations to set subtype and pathotype-specific international standards to better control H5 and H7 outbreaks, as these subtypes might cause epidemics of huge proportions, in different poultry species [1]. On the other hand, AIV of the H9N2 subtype are not subject to specific international control measures, as they are considered of low-pathogenicity for poultry and their subtype has never mutated to the highly pathogenic form.

Genetically, distinct H9N2 lineages have been described, the G1 being probably the most widespread. In fact, H9N2 viruses of the G1 lineage emerged in Hong Kong in 1997 [2], and steadily spread to the rest of Asia, reaching the Middle East [3] and North Africa [4–7], by the year 2000 and 2006, respectively. In 2016, the virus severely affected Morocco and, for the first time, was reported in sub-Sahara Africa, being reported in Burkina Faso, in West Africa [8]. In many of these regions, the virus has become an endemic presence notwithstanding the implementation of vaccination, increased biosafety and surveillance measures, and is frequently associated with moderate-to-high mortality in broilers and long-lasting drops in egg production in layers and breeders [4, 5, 9].

Several experimental studies have tried to reproduce the clinical presentations observed in the field, but mortality and severe respiratory signs were rarely observed unless the infection was artificially coupled with secondary pathogens such as *Escherichia coli*, *Mycoplasma gallisepticum*, *Staphylococcus aureus*, *Haemophilus paragallinarum*, *Chlamydia psittaci*, *Ornithobacterium rhinotracheale* and live infectious bronchitis virus vaccine [10–15] or in the presence of an immunocompromised flock [16]. In our opinion, these data together with the lack of ad hoc restrictive control measures and in-depth diagnostic analyses during outbreaks, have somehow mitigated the perception of H9N2 viruses as primary pathogens of poultry.

Although evidence from the field clearly indicates the ability of H9N2 viruses to induce pathology at the level of the oviduct [17], only few studies have investigated the tropism of these agents for this organ, in a controlled experimental setting [18–21].

Wang et al. proved in fact, that a H9N2 virus of the Y280 lineage isolated in the Shangxii province in 2011, could replicate at high titers in the oviduct, trigger apoptosis and cause a decrease in the number of laid eggs in the first week post infection and a reduction in egg-shell thickness [18, 20].

Inspired by this work, we decided to study the acute and chronic impact of a G1-lineage H9N2 virus on the performance of Hy-Line Brown hens at the peak of lay, to fully elucidate the etiology and pathogenesis of the disease in commercial layers.

## Materials and methods

### Animals and ethics

For this study, 70 Hy-Line Brown hens were bought from a local farm at the peak of lay (28 weeks of age) and housed in negative, high efficiency particulate air filtered poultry isolators, at the facilities of the Istituto Zooprofilattico Sperimentale delle Venezie. At the farm, birds were vaccinated against infectious bronchitis, Newcastle disease, infectious laryngotracheitis, Salmonella enteritidis, Marek’s disease, Infectious bursal disease, avian encephalomyelitis, avian metapneumovirus, coccidiosis, egg drop syndrome and fowlpox. Birds received feed and water ad libitum. Animal experiment procedures were conducted in strict accordance with the Decree of the Ministry of Health n. 26 of 4 March 2014 on the protection of animals used for scientific purposes, implementing Directive 2010/63/EU, and approved by the Institute’s Ethics Committee (n. 5/2014). Before the experiments, the birds were given a 4-weeks period to ensure acclimatization to the isolators, and the normalization of laying performances. Eggs were collected throughout the study, three times a day. Birds were screened for the presence of antibodies against type A Influenza virus nucleoprotein by using a commercial ELISA test (ID Vet, Montpellier, France) and resulted negative.

### Virus

A low pathogenicity AI (LPAI) virus of the H9N2 subtype belonging to the G1 lineage [22], A/chicken/Israel/1163/2011 was isolated at the Kimron Veterinary Institute from swabs collected during surveillance activities, in Israel [23]. A viral stock was produced by inoculating 9-to-11-day-old embryonated specific pathogen free (SPF) hen’s eggs, via the allantoic cavity and was subsequently titrated according to the Reed and Muench method [24], to determine the embryo infectious dose 50% (EID_50_). The stock was screened to exclude the presence of bacterial agents and common viruses of poultry as previously described [25]. The low pathogenicity of the virus had already been confirmed in a previous experimental study by calculating the intravenous pathogenicity index [26] that resulted to be 0. The nucleotide sequence of the hemagglutinin gene was made available in the GenBank database under the Accession Number JQ973659 by Davidson et al. [23]. The virus stock used in this studies had a history of three passages in embryonated chicken eggs.

### Experimental design

Birds were challenged via the oro-nasal route with a dose of 10^6^ EID_50_/0.1 mL of virus. All experimental infections were carried out on birds that were lightly sedated, injecting intramuscularly 150 µL of tiletamine and zolazepam, to prevent that the challenge inoculum was either sucked in or sprayed out by the animal. Negative control groups were sham infected reproducing the challenge protocol of sedation but administering 100 µL of negative allantoic fluid.

For the collection of organs birds were sedated with 300 µL of tiletamine and zolazepam via the intramuscular route and humanely killed via an intravenous administration of 200 µL of tetracaine.

In all of the experiments, birds were monitored twice a day to record the manifestation of clinical signs.

#### Experiment 1—Evaluation of clinical data and oviposition

In this experiment, 10 layers were challenged in the H9-group, and 10 birds served as negative controls, in the K-group. In this study, birds were only swabbed in the trachea once, on day 4 pi to ensure infection was ongoing. We kept handling to a minimum, to avoid disturbance of the animals and the interference of confounding factors in the assessment of laying performances. Eggs were collected, counted and evaluated in terms of shape, color and shell thickness using an analog micrometer with a sensitivity of 0.01 mm (Baxlo^®^ Precision, Spain). All of the birds were euthanized on day 80 pi to collect the oviduct and evaluate the histological architecture of the organ.

#### Experiment 2—Determination of organ colonization and shedding

In this experiment, 28 layers were challenged in the H9-group, and 12 birds served as negative controls, in the K-group. On days 4, 6, 10 and 18 pi, seven and three birds were randomly selected and euthanized in the H9 and K groups, respectively. Each of the seven birds in the H9-group, was necropsied and the oviducts were collected for virological, bacteriological and histological analyses, while for 4/7 of these animals a more comprehensive set of tissues was analyzed including, lungs, duodenum, cecal tonsils, spleen, kidney and brain, to evaluate the tissue tropism of the challenge virus. All of the birds in this experiment were sampled for tracheal swabs on day 4 pi, to confirm that the challenge had been followed by an infection. To evaluate the viral shedding, we randomly selected nine animals in this group and collected tracheal and cloacal swabs on day 2, 4 and 6 pi.

#### Experiment 3—Viremia

In this experiment, 10 birds were challenged to evaluate whether viremia played a role in the spread of the virus to extra-respiratory and extra-digestive tissues. Birds were swabbed in the trachea on day 2 pi, to ensure infection was ongoing. On days 2 and 3 pi, five birds were sedated with 300 µL of tiletamine and zolazepam via the intramuscular route and euthanized by exsanguination, via cardiac puncture to collect as much blood as possible and test both blood cells and plasma for the presence of virus RNA.

### Virus detection and quantification

Organ samples were weighed and homogenized, in a solution of phosphate buffered saline (PBS) and antibiotic–antimycotic (penicillin, streptomycin and amphotericin B). Homogenates were clarified by centrifugation at 10 000* g*, for 10 min at 4 °C, and stored at −80 °C.

We collected blood at a 1:1 ratio with Alsever’s solution to prevent coagulation and clarified 50 mL by centrifugation at 2000* g* for 30 min, at 4 °C to collect pelleted blood cells. Subsequently, we centrifuged the supernatant at 27 000* g* for 2 h at 4 °C, and irrespective of the presence of a visible pellet, the bottom of the tube was washed with 1 mL of PBS, and the collected fluid was stored at −80 °C.

Tracheal and cloacal swabs were eluted into 0.5 mL and 1 mL, respectively of PBS containing antibiotics. Viral RNA was extracted from 100 mL of either clarified-and-ultracentrifuged blood, tissue homogenates or pelleted blood cells, using the commercial NucleoSpinRNA kit (Macherey–Nagel, Duren, Germany) for manual extraction and from 50 µL of PBS containing a swab suspension, using the Ambion MagMax-96 AI-ND Viral RNA Isolation kit for the automatic extractor.

AIV quantitative real time RT-PCR (qRRT-PCR) was performed using the published probe and primers targeting the avian gene M from Spackman et al. [27].

For qRRT-PCR assays of blood and organs, a standard curve was generated using triplicates of ten-fold serial dilutions of in vitro transcribed RNA to obtain the quantification of the samples expressed in copies/µL. The in vitro transcribed RNA was generated using the MEGAscript T7 transcription Kit (Ambion^®^, Austin, USA) with the H7N1 subtype A/ostrich/Italy/984/2000 strain as a template.

The AIV standard curve was carried out for each run together with the samples. All tests were performed on the Bio-Rad CFX96 real-time PCR system and analyzed using the Bio-Rad software.

The sensitivity of the method, defined by the lowest concentration of the virus detected in all of the replicates in one RRT-PCR assay, was 10^4.1^ copies/µL for in vitro transcribed RNA.

All of the quantitative results were calculated as copies/µL equivalents and then normalized as copies/0.1 g of organic material, as all of the samples were weighed before processing.

Titration of infectivity was carried out by a standard tissue culture infectious dose 50% (TCID_50_) assay using MDCK cells. The presence of viral infection was determined at 72 h pi, by optical assessment of cytopathic effects coupled with a hemagglutination assay (HA). The titer was determined by the Reed and Muench method [24]. The limit of detection was of 20 TCID_50_/0.1 g of tissue.

### Differential bacteriological and virological analyses

As both primary and secondary bacterial colonization of the oviduct can occur in birds contributing to abnormal eggs in shape, color and size, we carried out bacteriological examination on oviduct specimens that had resulted positive for the presence of influenza A virus by RRT-PCR to investigate the presence of relevant bacteria (e.g. *E. coli*, *Gallibacterium* spp., *Enterococcus* spp., *Streptococccus* spp., *Pseudomonas* spp.). Moreover, specific PCRs for *Mycoplasma synoviae* and *galllisepticum* were performed from oviducts in order to rule out the onset of abnormal eggs due to mycoplasma infection.

Briefly, oviduct swabs were aseptically collected from each bird and the site of sampling was the same in all oviducts, thus a sterile cotton swab was scrubbed for 5 s on the oviductal mucosa between the infundibulum and magnum portions. Then, swabs were aseptically removed from the tissues and immediately put into an Amies transport medium for bacteria isolation (Copan Innovation, Brescia, Italy), thus these biological specimens were sent to the laboratory for standard microbiology examinations. Swabs were promptly inoculated into solid and liquid media for bacteria isolation such as Blood Agar plates, Mac Conckey plates, BEA plates (Bile Esculin Agar) and heart infusion broth (HIB) and cultivated at 37 °C under 5% CO_2_ environment for 24 h. After incubation, plates were checked for visible bacteria growth and in case of presence of colonies further standard bacteriological analyses were carried out for bacterial identification. On the contrary, if no colonies were present on agar surface and the HIB broth presented turbidity, some drops of the broth cultures were inoculated in new and sterile Blood Agar, Mac Conckey and BEA plates, which were incubated in the same physical conditions for further 24 h. Swabs for *Mycoplasma* spp. detection were submitted for DNA extraction by MagVetTM Universal isolation kit (Life Technologies, Carlsbad, CA, USA) and Microlab STARlet (Hamilton Robotics, Reno, NV, USA); *M. synoviae* was investigated through a Real Time PCR targeting the 16S-23S gene as described by Raviv and Kleven [28] with slight modifications. The end-point PCR for *M. gallisepticum*, targeting the MGC2 gene, was performed as described by Garcia et al. [29] with slight modifications.

Moreover, since IBV and NDV viruses can invade the oviduct and cause salpingitis in avian species, we carried out differential virological examinations on the oviductal swabs.

Swabs were processed for the extraction of RNA as described in the previous section. We performed a RRT-PCR targeting the 5′-untranslated region of the IBV genome according to Callison et al. [30] and a RRT-PCR targeting the NDV RNA-dependent RNA polymerase gene as previously reported [26].

### Histopathology and immunohistochemistry (IHC)

Organ samples were fixed in 10% neutral buffered formalin for 48 h, routinely processed for histology and 4-μm sections were stained with hematoxylin and eosin (HE). The infundibulum, magnum and uterus were examined for the presence of: (a) heterophilic infiltrate; (b) lymphocytic and plasmacytic infiltrate; (c) the presence of hemorrhage and/or edema; (d) necrosis; (e) the presence of hemorrhage and/or cell debris within the lumen. For each of these pathological features, a score was given as follows: 0 absence of the feature, 1 mild, 2 moderate, 3 severe. For each section scores were averaged to obtain a mean histopathology score. Mean scores were averaged for each oviduct section and sampling time.

H9N2 virus qRRT-PCR positive samples were tested by IHC and a score was assigned to each section as follows: 0 no staining, 1 small clusters of positive cells, 2 multiple accumulations of positive cells and 3 extensive staining. IHC was performed on 3-µm sections, using an automated immunostainer. For each oviduct section and sampling time scores were averaged. The antigen retrieval was performed using the commercial ready to use solution Protease 2 (Roche, Germany), at 36 °C, for 12 min. The slides were incubated with the monoclonal antibody clone 1331 (Biodesign, USA) targeting the nucleoprotein of influenza type A viruses applied at 1:1500 dilution, for 80 min at room temperature. An ultraView Universal DAB Detection Kit (Roche, Germany) was used as detection system. A positive control consisting of a lung of an Influenza A positive chicken and a negative control consisting of a kidney of an Influenza A negative chicken were included in each run. All of the tested tissues were also incubated with the negative control antibody MG2a-53 (ab18415, Abcam) matching the primary antibody species and isotype.

### Virus binding assay

H9N2 virus concentration and purification were carried out according to Hutchinson and Fodor [31].

Inactivation and labelling of the challenge virus were done according to van Riel et al. [32]. Briefly, inactivation was achieved by dialysis against 0.1% formalin for 3 days. After inactivation, the virus was dialyzed against PBS. We labelled the inactivated virus through an incubation of 1 h at room temperature with a 1:1 (v/v) 0.5 mol/L bicarbonate buffer solution (pH 9.5) containing 0.1 mg/mL of fluorescein isothiocyanate (FITC) (Sigma-Aldrich, USA). The virus was dialyzed in PBS to release unbound FITC, using Pur-A-Lyzer tubes (Sigma-Aldrich, USA), for 6 h.

Birds from negative control groups were selected as donors of tissues to study the binding preference of the challenge virus. The virus histochemistry (VHC) technique was performed on the infundibulum, magnum, isthmus and uterus according to the protocol developed by van Riel et al. [32]. Briefly, 3-µm sections were deparaffinized and hydrated with graded alcohols. Incubation with the labelled virus was carried out overnight, at 4 °C with either 5, 50 or 100 hemagglutinin units (HAU). To visualize the bound virus, slides were incubated with a monoclonal antibody conjugated with horseradish peroxidase targeting the FITC molecule labelling the virus. The signal was amplified with a tyramide signal amplification system (Perkin Elmer, MA, USA). Peroxidase was revealed with 3-amino-9-ethyl-carbazole (AEC) (Sigma-Aldrich, USA). Tissues were counterstained with hematoxylin and embedded in glycerol.

### Statistics

The non-parametric Mann Whitney test was used to compare the virus load of tracheal and cloacal swabs, and to establish whether virus loads in the oviducts were significantly different from those reported in the rest of the organs. Oviposition, eggshell thickness, and numbers of misshaped and discoloured eggs were expressed as daily means for each group. We compared the weekly means among groups using the Wilcoxon matched-pairs signed-ranks test, a two-tailed *P* value. Statistical analyses were carried out using the GraphPad Prism 5.0 software (GraphPad Prism, GraphPad Software, La Jolla CA, USA) and the significance level was set at *P* < 0.05.

## Results

### Experiment 1—Evaluation of clinical data and oviposition

Before the challenge, all of the animals looked healthy and did not display egg-eating behavior. During the 4 weeks of adaptation, we recorded the number, shape, color and shell thickness of eggs, and all parameters resulted to be within the normal ranges of the commercial line.

**Figure 1:**
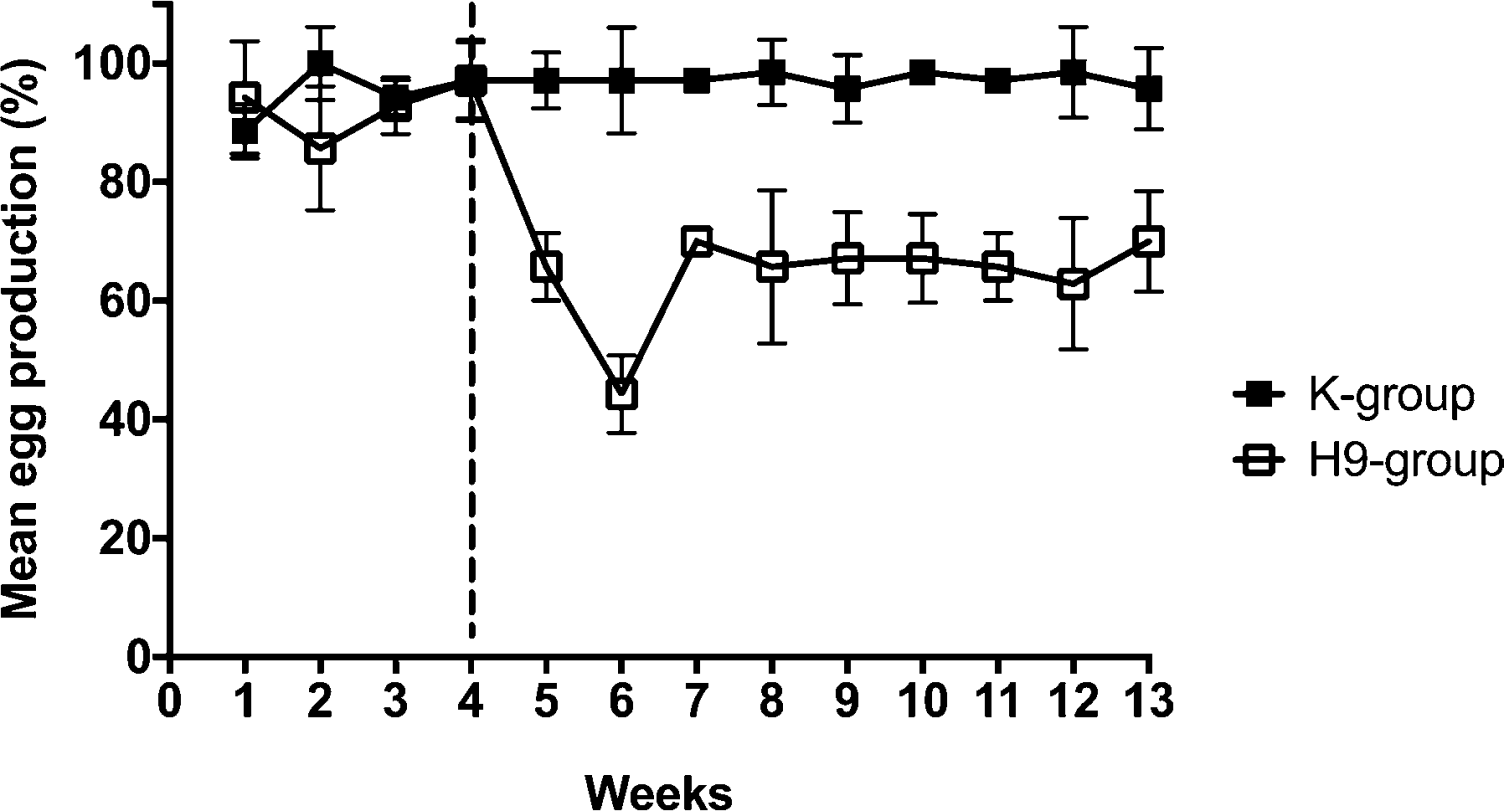
**Laying performance of challenged and control birds in experiment 1.** Eggs were collected throughout the experiment, three times a day, keeping handling of animals to the minimum, in order to avoid the occurrence of stressful events. We compared the egg production between challenged and control birds on a weekly basis, adopting the Wilcoxon matched-pairs signed-ranks on daily mean values for each group. Significance level was set at *P* < 0.05. Data are shown here as mean ± standard deviations. The differences in the weekly egg production between the two groups were significant (*P* < 0.05) for 9 weeks after the challenge.

All of the challenged birds shed viral RNA via the tracheal route on day 4 pi and showed signs of mild depression, ruffled feathers and a transitory arched posture, while we recorded conjunctivitis in approximately one-third of the layers. Birds started showing signs of the disease on day 3 pi and gradually recovered by day 6–7 pi. To compare egg production between groups on a weekly basis, daily values were averaged (Figure 1) and are here expressed as laying rates (%), considering a production of 10 eggs/10 hens/day as a hypothetical value of 100%. Before the infection, all of the layers in the H9 and K groups laid eggs scoring a mean production of 93.0% and 95.0%, respectively and we did not observe any statistical difference between the two groups.

In the H9-group, laying rates decreased to 65.7% and 44.3% 1 and 2 weeks after the challenge, respectively, while from week 3–9 pi values plateaued in the range of 62.8–70.0%. After the sham infection, birds in the control group recorded laying performances in the range of 95.7–98.5%. Statistical analyses indicate that the difference in egg production between the two groups was significant (*P* < 0.05) for 9 weeks after the challenge.

Occasionally, we observed discoloration and changes in the shape of eggs in the H9-group, but we did not find these features to be statistically different when compared with the K-group. The individual shell thickness fluctuated widely within and between groups, irrespective of the time of challenge. We found no statistical evidence that the replication of the virus related to a change in the thickness of the shell.

### Experiment 2—Determination of organ colonization and shedding

In 56%, 78% and 60% of the birds the cloacal swabs resulted positive on days 2, 4 and 6 pi, respectively (Additional file 1), while all of the birds shed virus RNA via the trachea at each sampling time. We compared the average Ct values of cloacal and tracheal swabs and observed that the virus load in the trachea was significantly higher than in the cloaca, on days 2 and 4 pi.

We evaluated the viral colonization of organs carrying out three complementary assays, namely the detection of the virus M gene by qRRT-PCR, isolation and titration of the virus in MDCK cells by TCID_50_ and the detection of the virus NP protein by IHC.

The qRRT-PCR detected virus RNA in all of the organs that are commonly assumed to be the target of LPAI viruses, namely the duodenum, the cecal tonsil or the lungs in 83% of the birds, throughout the study. Moreover, at least one of the four sections of the oviduct was positive by qRRT-PCR in 71%, 57% and 14% of the layers on days 4, 6 and 10 pi, respectively (Table 1).

**Table 1:** **Virus detection in the organs of birds of experiment 1 by means of RRT-PCR, IHC and isolation in MDCK cells**

Day post-infection	4			6			10		
	RRT-PCR pos/tot (%)^a^	IHC pos/tot (%)	TCID_50_ pos/tot (%)	RRT-PCR pos/tot (%)	IHC pos/tot (%)	TCID_50_ pos/tot (%)	RRT-PCR pos/tot (%)	IHC pos/tot (%)	TCID_50_ pos/tot (%)
Oviduct	5/7 (71)	2/7 (29)	3/7 (43)	3/7 (57)	3/7 (43)	3/7 (43)	1/7 (14)	1/7 (14)	0/7 (0)
Intestine	4/4 (100)	0/4 (0)	1/4 (25)	2/4 (50)	0/4 (0)	0/4 (0)	0/4 (0)	0/4 (0)	0/4 (0)
Cecal tonsil	2/4 (50)	0/4 (0)	1/4 (25)	2/4 (50)	0/4 (0)	0/4 (0)	0/4 (0)	0/4 (0)	0/4 (0)
Kidney	1/4 (25)	0/4 (0)	0/4 (0)	2/4 (50)	0/4 (0)	0/4 (0)	0/4 (0)	0/4 (0)	0/4 (0)
Spleen	1/4 (25)	0/4 (0)	0/4 (0)	2/4 (50)	0/4 (0)	0/4 (0)	1/4 (25)	0/4 (0)	0/4 (0)
Lung	3/4 (75)	0/4 (0)	2/4 (50)	3/4 (75)	0/4 (0)	0/4 (0)	3/4 (75)	0/4 (0)	0/4 (0)
Brain	1/4 (25)	0/4 (0)	0/4 (0)	0/4 (0)	0/4 (0)	0/4 (0)	0/4 (0)	0/4 (0)	0/4 (0)

pos: positive, tot: total.

^a^Percentage of positive animals; oviducts were considered positive when at least one section of the organ tested positive.

Let aside the anatomical district of the oviduct, we detected the virus RNA in organs beyond the digestive and respiratory tracts, namely in the kidney, spleen and brain of 25% of the layers on day 4 pi, and in the kidneys and spleens of 50% of the birds on day 6 pi.

**Figure 2:**
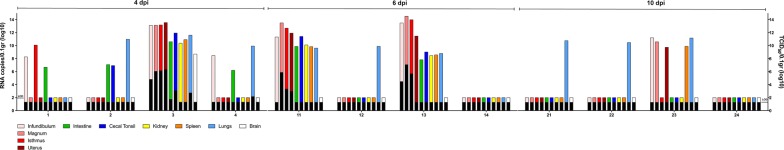
**Virus loads in organs collected from birds in experiment 2, on days 4, 6 and 10** **pi.** Virus colonization of organs was assessed and quantified by means of qRRT-PCR and TCID_50_, in MDCK cells. Colored and black columns represent the number of virus RNA copies and TCID_50_/0.1 g of tissue, respectively. Limits of detections for each scale are indicated by lateral bars. Values are shown on an individual basis, as indicated by the bird ID number on the X axis. LOD: limit of detection, inf: infundibulum, ma: magnum, is: isthmus, ut: uterus.

On day 10 pi, one out of four birds had virus RNA in the spleen (Figure 2). None of the organs collected on day 18 pi was positive by qRRT-PCR.

qRRT-PCR positive samples were further titrated to evaluate the infectivity of the virus. On days 4 and 6 pi, we recorded positive TCID_50_ values in the oviduct of 43% of the birds, while the organs of the respiratory and digestive tracts were positive in 50% and 25% of the birds respectively, only on day 4 pi (Table 1 and Figure 2). We did not observe cytopathic effects culturing kidney, brain and spleen samples.

Statistical analyses did not describe significant differences in virus load expressed as either genomic copies or TCID_50_ among the different organs.

**Figure 3:**
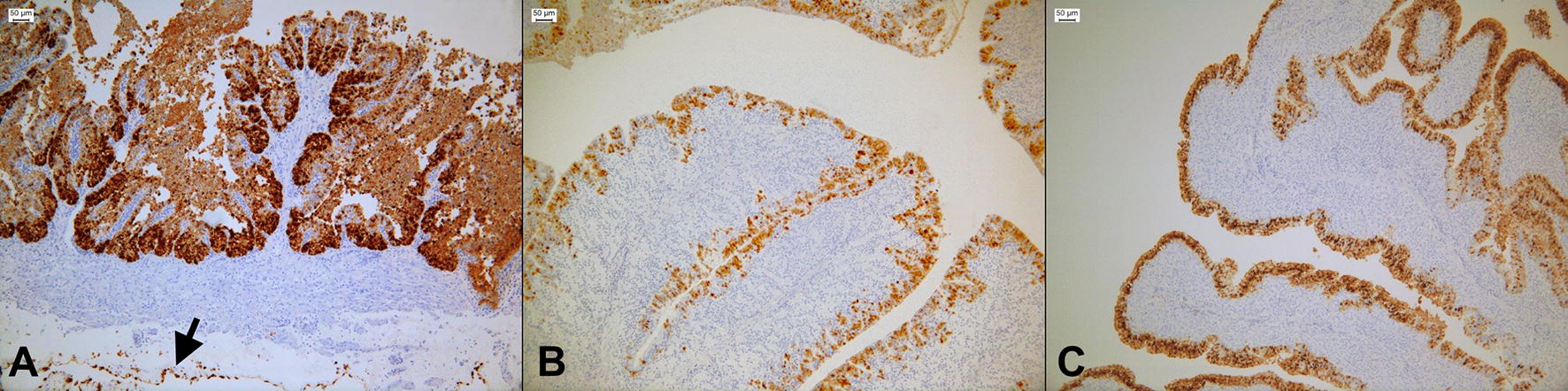
**Immunohistochemistry of the infundibulum, magnum and uterus of challenged birds in experiment 2.** Representative 3-µm sections of oviducts collected during the acute phase of infection, in experiment 1. Sections were incubated with a monoclonal antibody targeting the Influenza A virus nucleoprotein. Positivity was revealed with 3, 3-diaminobenzidine (DAB), forming a brown precipitate. **A** Strong positivity of an infundibulum collected on day 4 pi, showing severe necrosis and erosion of the epithelial mucosa. The arrow indicates positivity of the mesenchymal cells lining the serosa. Epithelial staining of **B** magnum and **C** uterus collected on days 6 pi.

IHC analyses revealed the presence of the NP protein only in the oviduct, while the rest of the organs were negative (Figure 3).

Colonization of the different sections of the oviduct was investigated comparing overall RRT-PCR positivity, TCID_50_ values, virus loads and IHC scores. The infundibulum resulted positive by RRT-PCR in a higher number of birds on days 4 and 6 pi (Table 2), while virus load (Additional file 2) and TCID_50_ values did not differ significantly among the oviduct sections.

**Table 2:** **Virus detection and histopathological damage in the different sections of the oviducts collected from day 4 to day 10** **pi**

Day post-infection	4			6			10		
	RRT-PCR positive/total (%)^a^	IHC score (%)^b^	Histopathology score (%)^c^	RRT-PCR positive/total (%)	IHC score (%)	Histopathology score (%)	RRT-PCR positive/total (%)	IHC score (%)	Histopathology score (%)
Infundibulum	5/7 (71)	0.85 ± 1.46 (29)	0.51 ± 0.63 (43)	4/7 (57)	0.85 ± 1.21 (43)	0.66 ± 0.77 (57)	1/7 (14)	0.14 ± 0.38 (14)	0.14 ± 0.19 (43)
Magnum	4/7 (57)	0.43 ± 0.79 (29)	0.17 ± 0.31 (29)	3/7 (43)	0.57 ± 1.13 (29)	0.11 ± 0.30 (14)	1/7 (14)	0.00 (0)	0.02 ± 0.07 (14)
Uterus	4/7 (57)	0.71 ± 1.25 (29)	0.00 (0)	3/7 (43)	0.14 ± 0.38 (14)	0.22 ± 0.35 (57)	1/7 (14)	0.00 (0)	0.00 (0)

In this table, we report the overall detection of virus RNA along with the presence of the virus nucleoprotein and the associated histological lesion, in the infundibulum, magnum and uterus of birds of the first experiment. RRT-PCR positive samples were tested by IHC and a score was obtained as follows: 0 no staining, 1 small clusters of positive cells, 2 multiple accumulations of positive cells and 3 extensive staining. Irrespective of RRT-PCR, all samples were examined for histological lesions taking into account the presence of: (a) heterophilic infiltrate; (b) lymphocytic and plasmacytic infiltrate; (c) hemorrhage and/or edema; (d) necrosis; (e) hemorrhage and/or cell debris within the lumen. For each of these pathological features, a score was given as follows: 0 absence of the feature, 1 mild, 2 moderate, 3 severe. Scores are expressed as mean values ± standard deviations.

N.D.: not done.

^a^Percentage of RRT-PCR positive animals.

^b^Percentage of IHC positive animals.

^c^Percentage of animals with histopathological changes.

The comparison of IHC scores among the different sections of the oviduct identified the infundibulum as the section recording the highest values (Table 2). IHC positivity was mostly limited to the nuclei and cytoplasm of the mucosal epithelia, even though we observed a specific and intense staining at the level of the serosal surface of the oviduct in 2/7 and 1/7 birds, on days 4 and 6, respectively.

### Experiment 3—Viremia

All of the birds shed virus RNA at the level of the trachea on day 2 pi, confirming that the challenge caused an infection. We did not detect virus RNA in either the clarified-and-ultracentrifuged plasma or the pelleted blood cells.

### Pathology and histopathology

Post-mortem examinations and histological analyses were performed on all of the euthanized birds in experiments 1 and 2. Results from experiment 2 will be first presented, to mirror the progression of the disease through time.

#### Experiment 2

None of the birds from the control group showed significant macroscopic changes in any of the examined organs.

Irrespective of the sampling time, we did not record any lesion in the lungs, kidneys, and brains of birds in the H9-group, while the spleens appeared slightly enlarged in all of the subjects, on days 4 and 6 pi.

Histological analyses indicated a mild-to-moderate congestion of the spleens and lungs collected on day 4, 6 and 10 pi, while the kidneys and brains appeared normal.

At the examination of the digestive tract, we observed the presence of fibrin and a yellowish exudate on the peritoneum of 4/7 and 5/7 of the birds, on days 4 and 6 pi, respectively.

Histological examinations described a mild superficial enteritis and a diffuse peritonitis with heterophilic and lymphoplasmacytic infiltration in the serosa of the duodenum of 2/4 subjects, on day 4 pi. Similarly, the cecal tonsils of these birds had a focal infiltrate of lymphoplasmacytic cells in the serosa. On day 6 pi, in 4/4 of the birds we recorded at the level of the duodenum a diffuse catarrho-erosive enteritis with a mild to moderate lymphoplasmacytic infiltration of the lamina propria, and in two of these animals we also observed a diffuse granulomatous peritonitis.

On days 10 and 18 pi, 2/7 and 3/7 of the birds respectively had yolk material and fibrin clots on the serosal surface of the organs in the abdomen, in association with a chronic granulomatous peritonitis at the level of the cecal tonsils.

**Figure 4:**
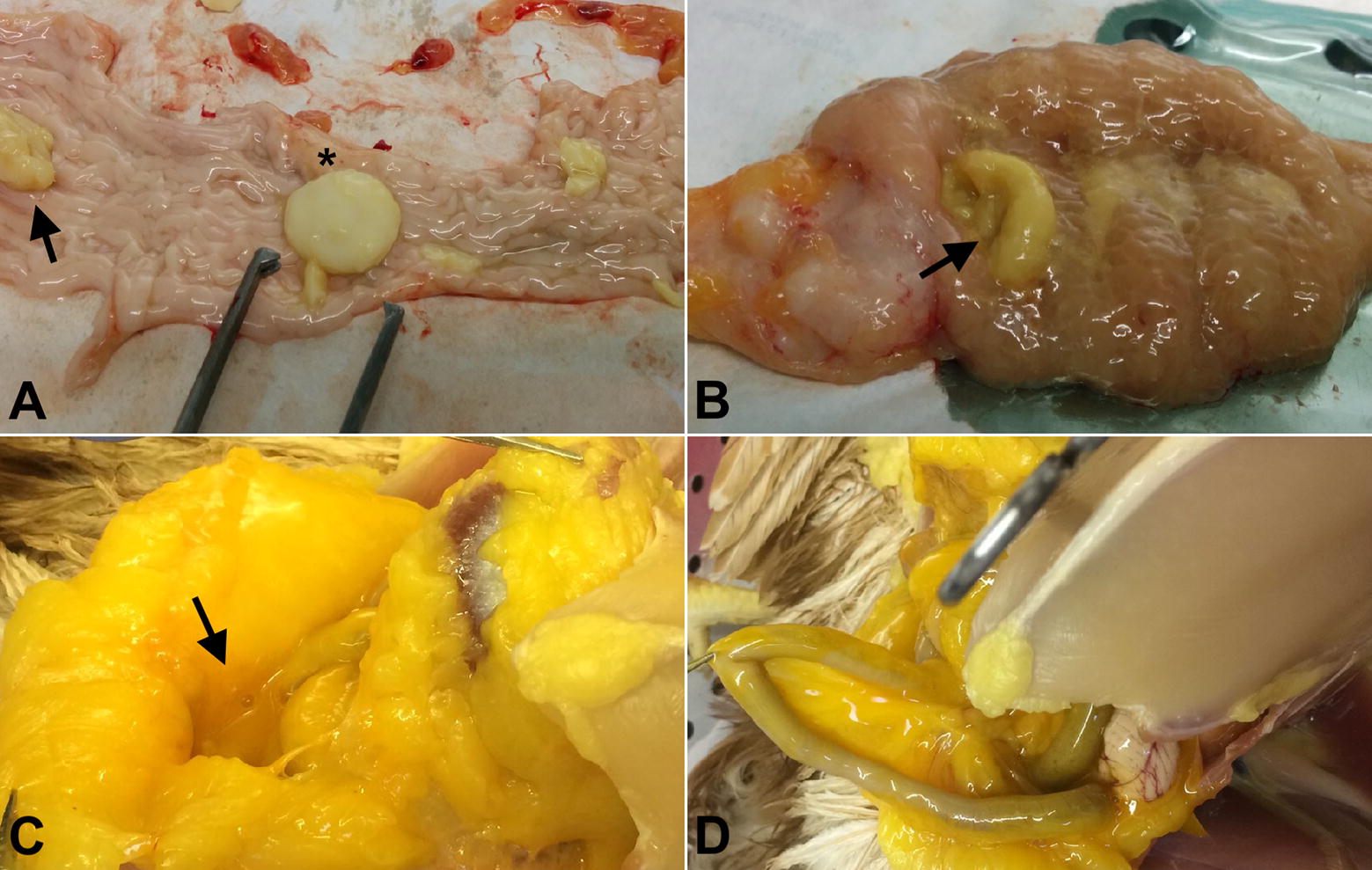
**Gross pathology of the oviduct and abdomen. A** A magnum collected on day 4 pi A defective egg and a fibrinous clot are indicated by an asterisk and an arrow, respectively. **B** A uterus collected on day 6 pi. The arrow indicates mucus and fibrin. **C**, **D** Egg yolk material in the abdomen of a bird necropsied on days 18 (**C**) and 80 pi (**D**).

The subjects with fibrin on the peritoneum had also oviducts with oedematous thickened walls, in which we found defective small eggs, abundant mucus, fibrin clots and purulent exudate, from day 4 up to day 10 pi (Figure 4).

**Figure 5:**
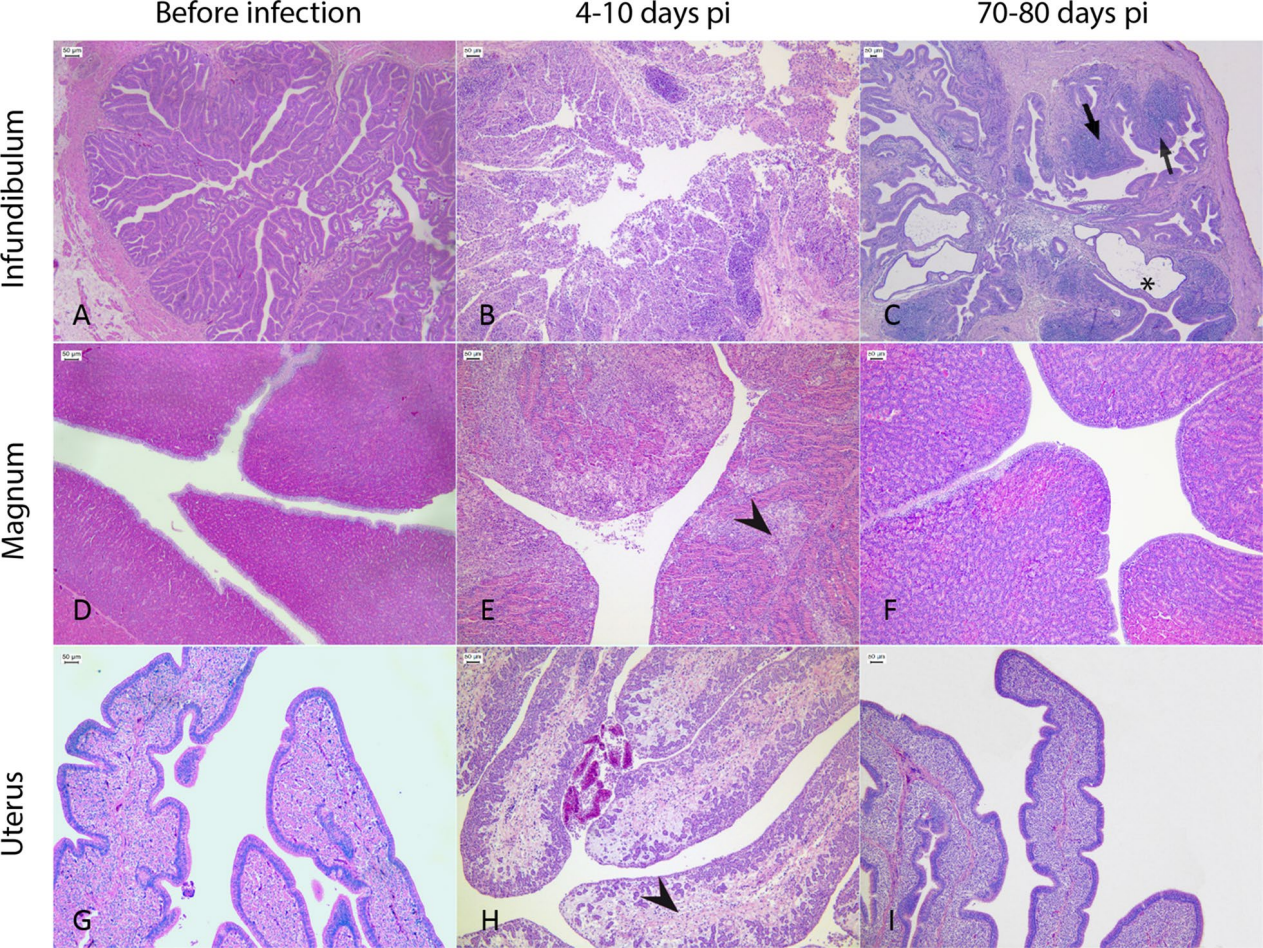
**Representative histological pictures of the infundibulum, magnum and uterus before, during and after the infection. A**–**I** All pictures were taken using a 10× objective from 4-μm sections stained with hematoxylin and eosin. **B** During the acute phase of infection (4–10 days pi), replication of the virus in the infundibulum was associated with the occurrence of severe necrosis, edema and granulomatous processes in the mucosa of the organ. **C** In the chronic phase of the disease (70–80 days pi), birds with egg yolk peritonitis showed a profound alteration of the infundibulum architecture due to the presence of ectatic glands (asterisk), hyperplastic lymphocytic aggregates (arrows) and thinning of the mucosa. **E**, **H** Lesions in the magnum and uterus were rarely observed during the acute phase of the infection and were limited to heterophilic necrosis and edema (arrow heads), respectively. **F**, **I** A full restoration of the tissue integrity was observed for the magnum and uterus in all subjects, after 70–80 days from infection.

Histological examination of the oviducts on day 4 pi indicated that in 2/7 of the animals, focal to multifocal erosion of the epithelium and a diffuse mild infiltrate of heterophils and macrophages were present in the mucosa of the infundibulum and in the serosa of the magnum. In one of these two animals, focal ulcerations of the mucosa were also described. On day 6 pi, in 3/7 birds, severe diffuse necrosis involving the full width of the tonaca mucosa, associated with edema and a granulomatous process were recorded at the level of the infundibulum (Figure 5), and in one of these subjects the lesions extended to the magnum and isthmus. In the other two subjects, the magnum, isthmus and uterus appeared normal. Ten days from challenge, 1/7 of the birds showed diffuse lymphoplasmacytic infiltration of the lamina propria, mild fibrosis and multifocal ectasia of the glands; in 4/7 of the birds a mild heterophilic and lymphoplasmacytic infiltration of the mucosa of the infundibulum was observed, and in two of these birds, a similar picture was observed at the level of the magnum, while the isthmus and uterus appeared normal. On day 18 pi, 3/7 of the birds had a diffuse lymphoplasmacytic infiltration in the mucosa of the infundibulum, with follicular organization, diffuse ectasia of the glands and interstitial fibrosis of the lamina propria. The histopathology score assigned to the infundibulum, magnum and uterus indicates the infundibulum as the most affected section of the oviduct from 4 to 10 days pi (Table 2).

Throughout the study, ovaries were productive as multiple follicles were visible during necropsies.

#### Experiment 1

Seven weeks after the challenge, given the chronic reduction in the number of laid eggs, we divided nine of the ten birds of the H9-group into nine additional isolators, to monitor the laying performances on an individual basis and relate it to the pathological and histopathological data. The separation of the layers allowed the identification of 3/10 non-laying birds, over a longer period of 4 weeks. On week 11 pi we euthanized and necropsied all of the birds and we observed egg yolk peritonitis in the three birds in which laying had stopped. Abundant yolk material filled the abdomen and the serosa appeared thickened and characterized by a strong yellow pigmentation. In these birds, the ovary had multiple follicles at different stages of maturation, indicating a normal function of the organ, and the oviduct appeared to be macroscopically normal.

The histological examination of the oviducts highlighted that in the three birds with egg yolk peritonitis, the infundibulum architecture was altered by the extensive presence of fibrosis, cystic glands and dysplasia of the mucosa; hyperplastic lymphocytic follicles and a diffuse lymphoplasmacytic infiltrate were also evident. The rest of the oviduct had no lesion (Figure 5; Table 3). We did not record any pathological aspect in the remaining animals, in which egg production had returned to normality.

**Table 3:** **Virus detection and histopathological damage in the different sections of the oviducts collected on day 18 and 80** **pi**

Day post-infection	18			80		
	RRT-PCR positive/total (%)^a^	IHC score (%)^b^	Histopathology score (%)^c^	RRT-PCR positive/total (%)	IHC score (%)	Histopathology score (%)
Infundibulum	0/7 (0)	0.00 (0)	0.29 ± 0.30 (57)	N.D.	0.00 (0)	0.32 ± 0.58 (40)
Magnum	0/7 (0)	0.00 (0)	0.43 ± 0.88 (57)	N.D.	0.00 (0)	0.10 ± 0.19 (20)
Uterus	0/7 (0)	0.00 (0)	0.09 ± 0.11 (43)	N.D.	0.00 (0)	0.00 (0)

In this table, we report the overall detection of virus RNA along with the presence of the virus nucleoprotein and the associated histological lesion, in the infundibulum, magnum and uterus of birds of the first experiment. RRT-PCR positive samples were tested by IHC and a score was obtained as follows: 0 no staining, 1 small clusters of positive cells, 2 multiple accumulations of positive cells and 3 extensive staining. Irrespective of RRT-PCR, all samples were examined for histological lesions taking into account the presence of: (a) heterophilic infiltrate; (b) lymphocytic and plasmacytic infiltrate; (c) hemorrhage and/or edema; (d) necrosis; (e) hemorrhage and/or cell debris within the lumen. For each of these pathological features, a score was given as follows: 0 absence of the feature, 1 mild, 2 moderate, 3 severe. Scores are expressed as mean values ± standard deviations.

N.D.: not done.

^a^Percentage of RRT-PCR positive animals.

^b^Percentage of IHC positive animals.

^c^Percentage of animals with histopathological changes.

### Bacteriology and differential virological analyses

To rule out the possibility that other microbiological agents played a role in the pathogenesis of the disease we carried out bacteriology examination, PCRs for *M. synoviae* and *gallisepticum*as well as virological analyses on oviductal swabs collected in experiment 2.

No bacterial growth was detected in the oviduct swabs of birds sampled on 4, 6 and 10 days pi, except for one subject on day 4 pi, in which we recorded the presence of few colony forming units (cfu) of *E. coli* and a low load of *Gallibacterium anatis*. *Mycoplasma synoviae* and *M. gallisepticum* were investigated through PCRs and all oviduct samples resulted negative for mycoplasmas. The presence of IBV and NDV virus RNA in the oviducts was ruled out, as all of the swabs were negative by means of RRT-PCR.

### Virus binding

The incubation of the labelled H9N2 virus with the four oviduct sections resulted in a heterogeneous binding profile.

**Figure 6:**
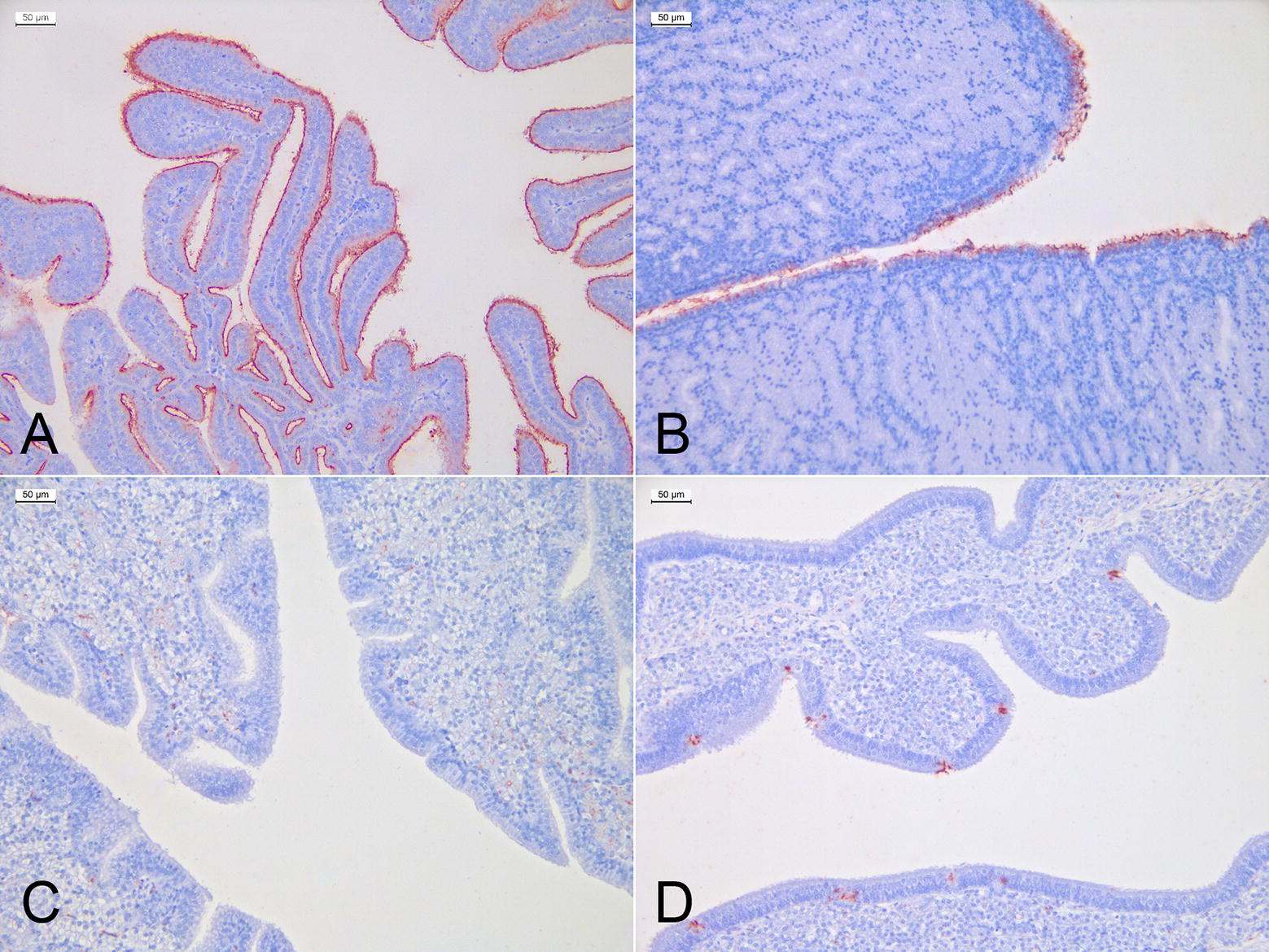
**Virus-histochemistry of the oviduct.** Red staining indicates the binding of the FITC-labelled virus to the tissues. Sections are counterstained with hematoxylin. **A** The infundibulum showed a highly specific and uniform positivity of the cilia at the level of the epithelium lining the fimbriae. **B** In the magnum, we observed a specific staining of the ciliated epithelium but intensity was lower compared with the infundibulum. **C** No attachment of the virus was observed at the level of the isthmus epithelium. **D** The uterus showed a scattered but specific attachment of the virus to the cytoplasm of epithelial goblet cells.

At the level of the infundibulum, the virus attached abundantly and with high specificity to the cilia of the epithelial cells lining the fimbriae (Figure 6). In the magnum, virus binding occurred at the level of the ciliated epithelium, and although positivity was highly specific, staining intensity was lower than the one recorded in the infundibulum. The isthmus showed no attachment of the virus to the epithelial cells while staining localized to granules within secretory glands in the lamina propria. The uterus showed a highly specific attachment of the virus to the cytoplasm of epithelial goblet cells.

Throughout the entire oviduct, the outer serosa was specifically and abundantly positive.

To ensure that the binding profile was not biased by the amount of virus used for the incubation step, three doses of 5, 50 and 100 HAU were used. The specificity of the staining was the same from 5 to 100 HAU in the infundibulum and magnum, while non-specific staining was slightly increased at the level of the isthmus and uterus (Additional file 3). Nevertheless, irrespective of the amount of virus used, positivity at the level of the epithelia was maintained.

## Discussion

In this study, we modelled a G1-lineage LPAI H9N2 infection in commercial layers, in order to reproduce and investigate the long-term drop in egg production frequently observed in the field.

Besides the expected self-limiting mild respiratory disease observed in each of the challenged birds, the H9N2 virus spread beyond the respiratory and digestive tracts and, in the absence of bacterial infections, robustly replicated in the oviduct of 62% of the hens during the first 4–6 days pi. In these birds, we noticed that the ovaries were productive, but in the lumen of the oviducts, there were abortive, defective eggs, mucous and fibrinous clots. In particular, according to both virological and histopathological data, the most affected section was the infundibulum, and in 29% of the birds in the first 4–6 days pi, the mucosal epithelium was extensively ulcerated and immunohistochemistry revealed diffuse positivity in the remaining epithelial cells. On the other hand, despite the evidence of viral replication in the remaining parts of the oviduct, the degree of inflammation was generally lower in the magnum, and practically absent at the level of the uterus. Interestingly, the proportion of animals with an H9N2 infection in the oviduct (62%) closely translated into a proportional drop in egg production during the first 2 weeks post infection, as the average number of laid eggs per day decreased from approximately 10.00 to 4.43 eggs/group, suggesting that the acute and subacute inflammation of the organ, in particular at the level of infundibulum, impaired the oviposition in each of the infected hens.

From 2 weeks up to 80 days pi, the number of eggs increased but reverted to only 65% of the pre-infection average production. Interestingly, the 3/10 layers that did not lay eggs 11 weeks pi had egg yolk material in the abdomen and normal ovaries with multiple follicles. Histological examinations linked the presence of yolk to a severe distortion of the architecture of the infundibulum, where we recorded thinning of the mucosa, diffuse presence of reactive plasmacytic aggregates and the atypical presence of ectatic glands in the lamina propria. Although we could not examine the birds beyond the eleventh week post-infection and throughout their entire commercial life, we reckon that the histological impediment of the infundibulum had become a chronic condition that could have permanently undermined the ability of this tissue to actively capture ova and convey them to the magnum. On the other hand, the rest of the birds completely recovered from the infection, showing no chronic lesion throughout the oviduct.

In light of these data, we speculate that the proportion of birds with severe histopathological changes in the mucosa of the infundibulum (29%) on days 4 and 6 pi closely related to the proportion of hens with chronic lesions (30%) on days 18 and 80 pi, suggesting a causal link between the two pathological processes.

Unfortunately, in our study we did not test the ovaries for virological and histopathological analyses, hence we missed important information that might have completed the pathogenetic picture. For this reason, we should not exclude that the drop in egg production, in particular during the acute phase of the disease, could have also been caused by a viral oophoritis.

The observed acute and chronic drops in egg production correlate with reports in the literature, in which acute declines range from 30 to 80% and chronic ones, although less frequently described, range from 10 to 25% [4, 18, 33–35]. In particular, in Morocco and in the Middle East, G1 virus infections have been associated with chronic declines up to 11–14 weeks after the beginning of the outbreaks [4, 17].

In the field, egg peritonitis is often considered of multifactorial etiology and one of the most important causes of mortality in layers and breeders [36]. Overfeeding, stress, genetics and improper lighting may all determine the occurrence of this syndrome, but in some cases, single factors such as viral infections of the reproductive tract (e.g. infectious bronchitis, Newcastle disease, HPAI viruses) can trigger the disease regardless of other managerial issues [37]. If bacterial agents as *E. coli* gain access to the peritoneum, either via reverse peristalsis along the oviduct or crossing of the intestinal barrier [38], the peritonitis becomes septic and more likely to bring the animal to a severe pathological state including lethargy, ascites, abdominal swelling and weight loss.

In light of this, and considering the good health *status* of birds at the end of the study, together with the granulomatous peritonitis and the altered architecture of the infundibulum, we speculate that the presence of yolk in the abdomen was the result of erratic oviposition [37] due to a chronic damage of the oviduct. According to these evidences, the observed pathological picture could fall under the definition of non-septic egg peritonitis.

The spread of this LPAI virus beyond the respiratory and digestive districts and its high tropism for the infundibulum called for an in-depth analysis of the virus phenotype. Firstly, we evaluated the timing and routes of viral spread to the organs by RRT-PCR analysis of both the cellular and non-cellular fractions of the blood but we could not detect virus RNA. Even though we consider these negative results as the possible consequence of both a suboptimal sensitivity of the molecular techniques applied and the limited sampling times, we should also consider that H9N2 viruses have been rarely detected in the blood of chickens [39, 40], either in the presence of a *S. aureus* co-infection [10] or after a combination of an intranasal and intratracheal challenge [41].

Although we did not detect virus RNA in the blood, in some subjects the spleens, kidneys, brain and the oviducts were RRT-PCR positive on days 4 and 6 pi, even though attempts to isolate the virus and detect it by IHC gave positive results only in the case of the oviducts.

We hence hypothesize that the virus might have spread to the oviduct either systemically or via alternative routes, either by ascending the cloaca, or by contact with the infected abdominal air sacs and ovary. The latter case would be in keeping with the replication of the virus in the mesothelial cells of the oviduct serosa and the strong binding of the labelled virus to these cells. Unfortunately, we have no sufficient evidence to clearly support or discard any of the aforementioned routes.

The other intriguing feature of this virus is the strong tropism for the oviduct tissues, and in particular for the infundibulum. This feature might be a strain-specific characteristic, depending on either the ability to spread systemically, the cleavability of the HA by unknown oviduct-specific enzymes [42], or the HA receptor binding preference that determines the attachment of the virus to the epithelia. Besides HPAI viruses, known to replicate in the reproductive tract of chickens [43], LPAI viruses have been associated with oophoritis and salpingitis of gallinaceous poultry but mostly in turkey breeders [44–46], while only few reports describe the colonization of the oviduct by non-H9 subtypes of LPAI viruses upon either natural or experimental infection [47–49].

There is no consensus regarding the distribution and type of sialic acids on the epithelia along the chicken oviduct. Wang et al. [19] found that the infundibulum was the only section of the oviduct to be lacking sialic acids to which both the lectins *Maackia amurensis* (MAA) and the *Sambucus nigra* (SNA) could attach, while in the rest of the oviduct MAA bound abundantly to the epithelium, hence revealing a prevalence of avian-like α-2,3 sialic acids. Mork et al. [50] reported a completely different binding profile for the infundibulum, in which both SNA and MAA II abundantly attached to the epithelium, while in the rest of the oviduct, they confirmed a significant higher expression of α-2,3 sialic acids binding to both MAA I and MAA II, a result in keeping with the data by Sid et al. [42]. The apparent contradiction of these data likely derives from the different types, brands and concentrations of lectins and revelation methods adopted in the two studies [51, 52]. To overcome these issues, we opted for an alternative approach, incubating the oviduct histological sections with the labelled H9N2 virus and revealing its attachment by means of immunohistochemistry. The results confirmed that this virus has a strong specific preference for the cilia of the infundibulum epithelium, while in the magnum and uterus the virus attaches less than in the infundibulum, and even fails to do so at the level of the isthmus. These data, together with the higher incidence and severity of infections observed in the infundibulum, indicate that the high affinity of this virus for the receptors expressed in this section of the oviduct might be one of the main etiological determinants of the disease.

In the two papers in which oviduct infections were investigated upon experimental challenges with recent H9N2 viruses circulating in the Shaanxi province, China [18, 19], the most affected sections were the magnum and the uterus, and in one study the infundibulum resulted to be negative by RRT-PCR. In one of these papers, a 60% drop in egg production in the first 7 days pi was associated with deposition of soft-shell eggs and a sudden decrease in the content of calcium in the eggshell, together with a reduction in the egg-shell thickness, confirming an overall preference of this virus for the chicken uterus [18]. These data and ours indicate that H9N2 viruses with a specific tropism for the chicken oviduct might have different anatomical preferences within the organ and this consistently translates into different pathological and productive scenarios.

In light of the laying performance, virological and pathological data, we believe that the experimental setting successfully reproduced the clinical disease, and the associated acute and chronic drops in egg production observed during G1-lineage H9N2 outbreaks in the field [4, 23]. Since we ruled out other microbiological agents as causes of salpingitis, the clinical picture associated with the replication of this virus in healthy chicken layers, allow us to think of it as an atypical pathotype of LPAI virus, capable of causing permanent economic losses, regardless of concomitant diseases in the flock. Under this perspective, we think this virus should be considered a primary pathogen of the oviduct, similarly to IBV, NDV and HPAI viruses [43, 53].

Although other papers have experimentally proven a causative relationship between the replication of H9N2 viruses in the oviduct of layers and changes in the quality and number of laid eggs [18, 19], their focus was limited to 1 week after the infection of SPF White Leghorn hens, and no microbiological examination was performed.

This paper is the first to offer an in-depth description of the pathogenic mechanism underlying the acute and chronic drops induced by a G1-lineage H9N2 virus in commercial layers.

To date, as opposed to HPAI viruses [54], LPAI viruses have never been isolated from the internal content of eggs [55]. Given the high tropism of this virus for the oviduct, further analyses will clarify the role of eggs and egg products in the transmission of H9N2 viruses in poultry and the potential threat posed by their consumption in humans.

The control of this non-notifiable disease in Asia, the Middle East, and North Africa has been mainly restricted to the use of vaccines, and yet H9N2 viruses have evolved antigenically [56] and spread to new geographic areas, causing huge economic losses to the poultry industry. H9N2 viruses have shown to be highly promiscuous and to be carriers of particularly advantageous genes for the emergence of LPAI reassortant strains with zoonotic potential, such as the H7N9 and H10N8 viruses [57]. Moreover, there are serological and virological evidences of poultry-to-human and poultry-to-pig transmission of H9N2 viruses in Southeast Asia and in the Middle East [58–60].

We are well aware that our results and the conclusions we draw should not be transposed blindly to other H9N2 viruses, but we believe this experimental setting shall be replicated with other strains circulating in the field, in order to build additional evidence in support of a change in the control of this disease.

## Additional files

**Additional file 1.** **Tracheal and cloacal shedding of virus RNA of birds, in experiment 2.** Shedding is expressed as both RRT-PCR Ct individual values and means ± standard deviations. Statistical was set at a *P* < 0.05. On days 2 and 4 pi, tracheal swabs had significantly higher Ct values than cloacal swabs (*P* < 0.00005).

**Additional file 2.** **Virus loads in oviducts collected on days 4, 6 and 10 pi, in experiment 2.** Shedding is expressed as both RRT-PCR Ct individual values and means ± standard deviations. Statistical was set at a *P* < 0.05. No significant difference was recorded among the different oviduct sections at any given sampling time.

**Additional file 3.** **Virus-histochemistry of the oviduct tract using 5, 50 and 100 hemagglutinating units of the H9N2 virus.** Red staining indicates binding of the FITC-labelled virus to the tissues. Sections were counterstained with hematoxylin. The specificity of the staining was the same from 5 to 100 HAU in the infundibulum and magnum, while non-specific staining slightly increased at the level of the isthmus and uterus at doses of 50 and 100 HAU. Irrespective of the concentration, the serosa stained consistently in all sections.

## Abbreviations

AIV: avian influenza viruses
LPAI: low-pathogenicity avian influenza
SPF: specific pathogen free
EID_50_: embryo infectious dose 50%
pi: post-infection
PBS: phosphate buffered saline
qRRT-PCR: quantitative real time RT-PCR
TCID_50_: tissue culture infectious dose 50%
HA: hemagglutination assay
IHC: immunohistochemistry
HE: hematoxylin and eosin
FITC: fluorescein isothiocyanate
VHC: virus histochemistry
CFU: colony forming unit
HAU: hemagglutinin units
MAA: *Maackia amurensis*
SNA: *Sambucus nigra*
L: leucine
D: aspartate
E: glutamate
A: alanine
I: isoleucine
