# Supplementary figures and images for: A G1-lineage H9N2 virus with oviduct tropism causes chronic pathological changes in the infundibulum and a long-lasting drop in egg production
Source: Vet Res. 2018 Aug 29;49:83. doi: 10.1186/s13567-018-0575-1 (PMC6116506; doi:10.1186/s13567-018-0575-1)

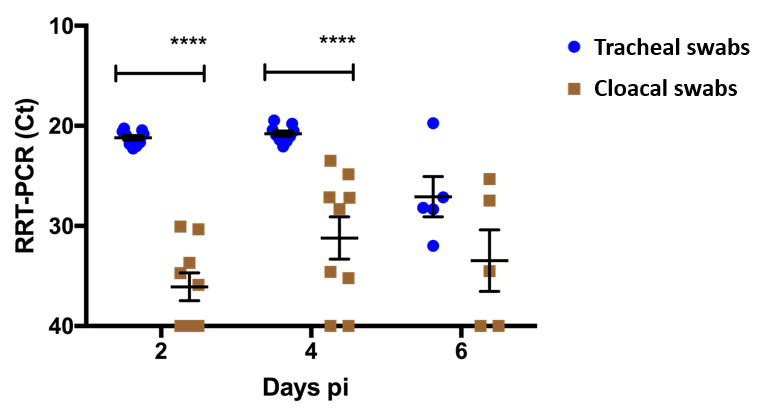

Supplement: Supplementary file 1 — Additional file 1. Tracheal and cloacal shedding of virus RNA of birds, in experiment 2. Shedding is expressed as both RRT-PCR Ct individual values and means ± standard deviations. Statistical was set at a P < 0.05. On days 2 and 4 pi, tracheal swabs had significantly higher Ct values than cloacal swabs (P < 0.00005). [file 13567_2018_575_MOESM1_ESM.tif]

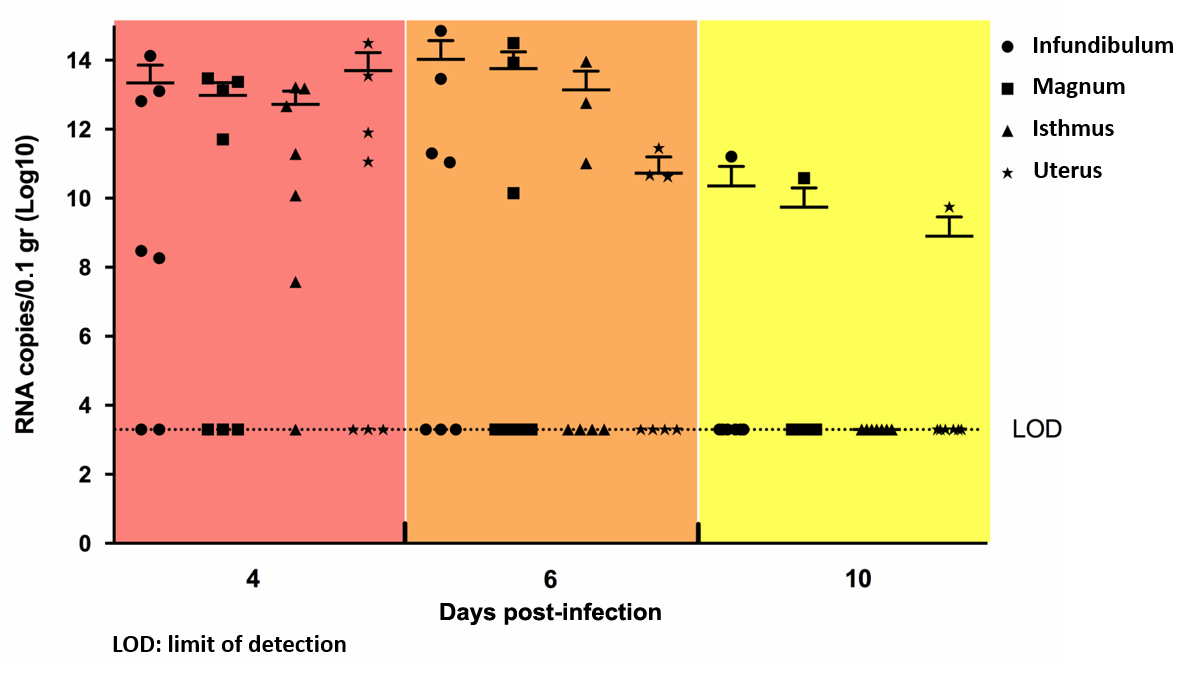

Supplement: Supplementary file 2 — Additional file 2. Virus loads in oviducts collected on days 4, 6 and 10 pi, in experiment 2. Shedding is expressed as both RRT-PCR Ct individual values and means ± standard deviations. Statistical was set at a P < 0.05. No significant difference was recorded among the different oviduct sections at any given sampling time. [file 13567_2018_575_MOESM2_ESM.png]

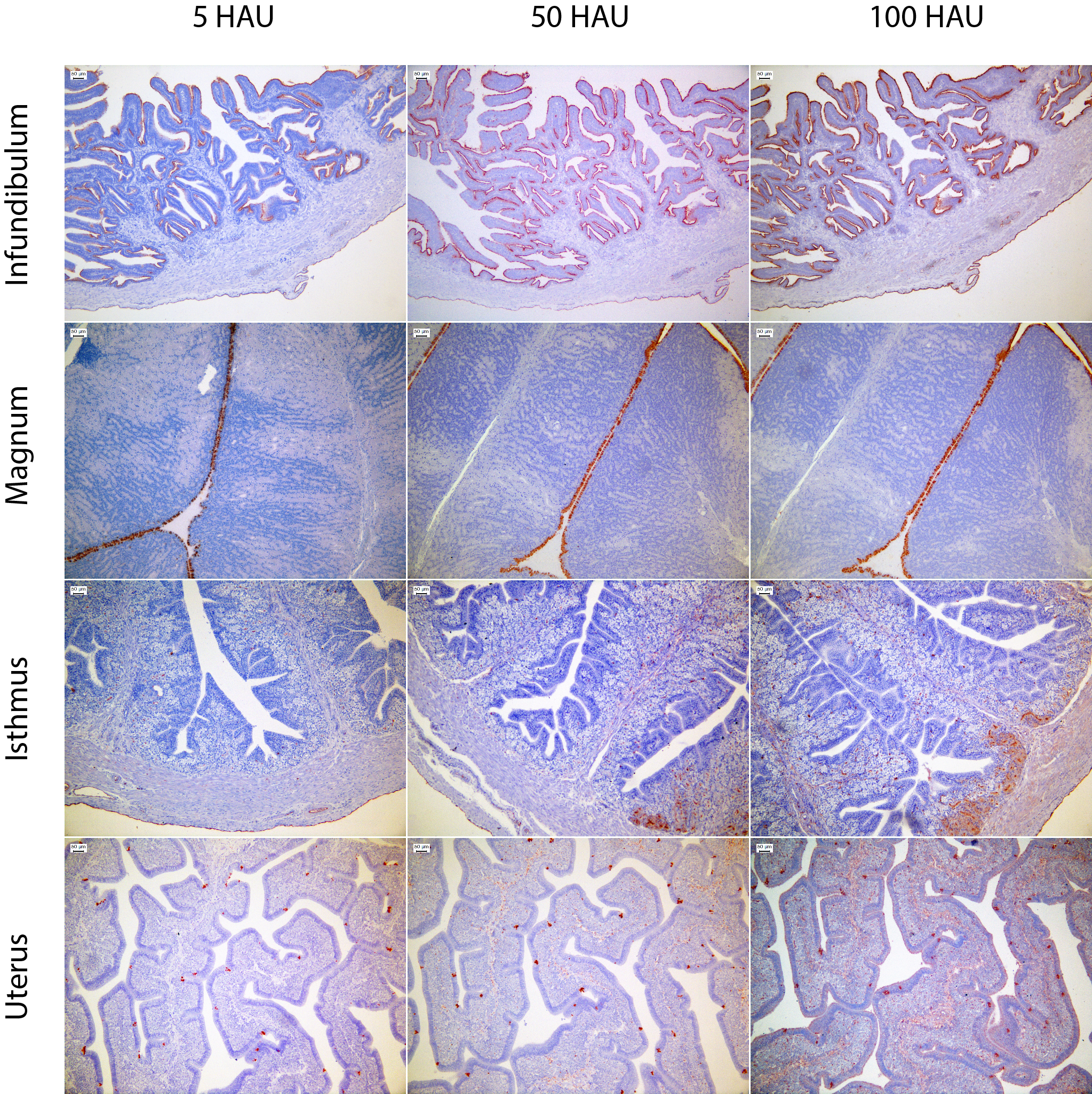

Supplement: Supplementary file 3 — Additional file 3. Virus-histochemistry of the oviduct tract using 5, 50 and 100 hemagglutinating units of the H9N2 virus. Red staining indicates binding of the FITC-labelled virus to the tissues. Sections were counterstained with hematoxylin. The specificity of the staining was the same from 5 to 100 HAU in the infundibulum and magnum, while non-specific staining slightly increased at the level of the isthmus and uterus at doses of 50 and 100 HAU. Irrespective of the concentration, the serosa stained consistently in all sections. [file 13567_2018_575_MOESM3_ESM.png]
